# Optimizing the MAC Protocol in Localization Systems Based on IEEE 802.15.4 Networks

**DOI:** 10.3390/s17071582

**Published:** 2017-07-06

**Authors:** Juan J. Pérez-Solano, Jose M. Claver, Santiago Ezpeleta

**Affiliations:** Departament d’Informàtica, Universitat de València, Avd. de la Universitat, 46100 Burjassot, Spain; jclaver@uv.es (J.M.C.); Santiago.Ezpeleta@uv.es (S.E.)

**Keywords:** fingerprinting, localization, MAC, multi-channel, wireless sensor networks

## Abstract

Radio frequency signals are commonly used in the development of indoor localization systems. The infrastructure of these systems includes some beacons placed at known positions that exchange radio packets with users to be located. When the system is implemented using wireless sensor networks, the wireless transceivers integrated in the network motes are usually based on the IEEE 802.15.4 standard. But, the CSMA-CA, which is the basis for the medium access protocols in this category of communication systems, is not suitable when several users want to exchange bursts of radio packets with the same beacon to acquire the radio signal strength indicator (RSSI) values needed in the location process. Therefore, new protocols are necessary to avoid the packet collisions that appear when multiple users try to communicate with the same beacons. On the other hand, the RSSI sampling process should be carried out very quickly because some systems cannot tolerate a large delay in the location process. This is even more important when the RSSI sampling process includes measures with different signal power levels or frequency channels. The principal objective of this work is to speed up the RSSI sampling process in indoor localization systems. To achieve this objective, the main contribution is the proposal of a new MAC protocol that eliminates the medium access contention periods and decreases the number of packet collisions to accelerate the RSSI collection process. Moreover, the protocol increases the overall network throughput taking advantage of the frequency channel diversity. The presented results show the suitability of this protocol for reducing the RSSI gathering delay and increasing the network throughput in simulated and real environments.

## 1. Introduction

Nowadays, the development of accurate indoor location systems is of great importance in the realization of a wide range of applications, such as: indoor navigation systems, location aware services, collaborative and autonomous robots, etc. The implementation of such systems using radio frequency signals is a challenge due to the physical properties of these signals and it has been treated from different point of views. As a result, a large quantity of research papers proposing distinct methods and algorithms can be found in the bibliography [1,2,3,4]. This work is focused on fingerprinting localization systems [5,6]. A main feature of this group of algorithms is the initial collection of RSSI values acquired at specific positions in the covered area. The RSSI measures are taken using a mobile radio transceiver that exchanges data packets with the infrastructure beacons. Once the database with all these measures is conformed, new users can locate themselves in this scenario comparing their own RSSI samples with the RSSI values in the database. However, continuous tracking of users requires the exchange of a burst of multiple data packets to gather the RSSI values at every new step. Moreover, as it is shown in references [7,8], the achieved location accuracy can be improved if the RSSI values are taken at both motes, (i.e., beacon and user), with different signal power levels and frequency channels. The total number of packets transmitted at every new position in this last case increases drastically. In addition, it represents a challenge because several users may be trying to access the medium to exchange packets with the same or with other beacons at the same time and in the same broadcast domain.

Wireless sensor networks (WSN) is a key technology in the development of location and tracking systems. WSN motes are simple low cost devices capable of performing wireless communications. Usually, the wireless transceivers integrated in WSN motes are IEEE 802.15.4 [9] compliant. This standard was conceived to support the design of low-rate personal area networks. In this kind of networks contention-based protocols are usually implemented at the medium access control (MAC) layer. In this context, the well-known carrier sense multiple access with collision avoidance (CSMA-CA) algorithm, proposed in the IEEE 802.15.4 standard, is the most common MAC protocol used in low-rate WSN. Nevertheless, contention-based protocols present serious restrictions in terms of maximum bandwidth and network throughput when the number of nodes and the transmission rate grows due to the increasing number of packet collisions [10,11,12]. On the other hand, time division multiple access (TDMA) MAC protocols can be considered as an alternative to implement the MAC layer. These protocols are more suitable for high-rate applications because the medium access is divided in time slots that are assigned to nodes individually, avoiding contention periods and packet collisions. However, the main drawback of TDMA [13] protocols is the tight clock synchronization that they require to identify the starting points of every time slot. The implementation of such time synchronization protocols in WSN is a great challenge due to the low resources and capabilities of the network nodes and the time uncertainty associated to packet transmission delays. 

Another approach that can be taken into account to increase the overall channel bandwidth is based on the use of different frequency channels. In this sense, reference [14] presents a protocol specifically designed for WSNs that assigns a frequency channel and a time slot to every mote, increasing significantly the network throughput. However, this protocol presents long delays in dense networks. In [15] authors propose a channel-hopping technique combined with contention-based access for packet transmissions. Nonetheless, the estimation of a suitable length for the schedule is not an easy matter and this fact affects the delivery latency. The work presented in [16] takes advantage of different frequency channels to provide a conflict-free schedule, but the channel assignment and the transmission scheduling needs complex algorithms, especially in dense networks. In [17] a tree network is split in separate smaller networks that make use of dedicated frequency channels, but the network topology does not consider mobile nodes. The protocol presented in [18] builds a tree and it allows that nodes at the same level in the tree compete to transmit packets within a shared slot. In [19] a centralized MAC protocol specifically designed for the implementation of indoor location systems is proposed. In this protocol, a special central node orchestrates the transmission of beacons to mobile nodes using a special trigger message. The main features of this protocol are: (a) the medium access is based on TDMA, assigning different slots to each node, (b) packets are transmitted using only one frequency channel, and (c) the network topology is restricted to the case where all the nodes are on the same broadcast domain, which limits the use of this approach on wide areas. Moreover, the use of TDMA requires strict time synchronization among different nodes. As a conclusion, it is observed that all these protocols do not meet the localization system requirements, since they deal with static or specific topologies and they introduce medium access protocols with tight time synchronization constraints, which in general are too complex for our purposes.

In this work, a first approach to implement the underlying location system infrastructure is based on the CSMA-CA protocol, as it is presented in the IEEE 802.15.4 standard [9]. However, due to the low performance that this approach provides, a new MAC protocol is proposed. This new protocol is adapted to the localization system requirements and its main objective is the reduction of the overall delay that appears during the RSSI sampling process. To achieve this end, the protocol has to cope with changes in the network topology and transmissions at different frequency channels. But it also has to provide a low computational cost, because it has to be implemented in motes with limited resources. The proposed protocol has been evaluated simulating different numbers of users and transmitted packets during the RSSI sampling process.

The rest of this paper is organized as follows. Section 2 is devoted to presenting an extensive analysis of the MAC protocol proposed in the IEEE 802.15.4 standard. Section 3 provides a new MAC protocol specifically tailored to the implementation of RSSI localization systems based on IEEE 802.15.4 networks. Results showing the performance improvement in terms of network throughput and data collection delay in simulated and real environments are presented in Section 4. Finally, concluding remarks are given in Section 5.

## 2. IEEE 802.15.4 MAC Analysis

**Figure 1:**
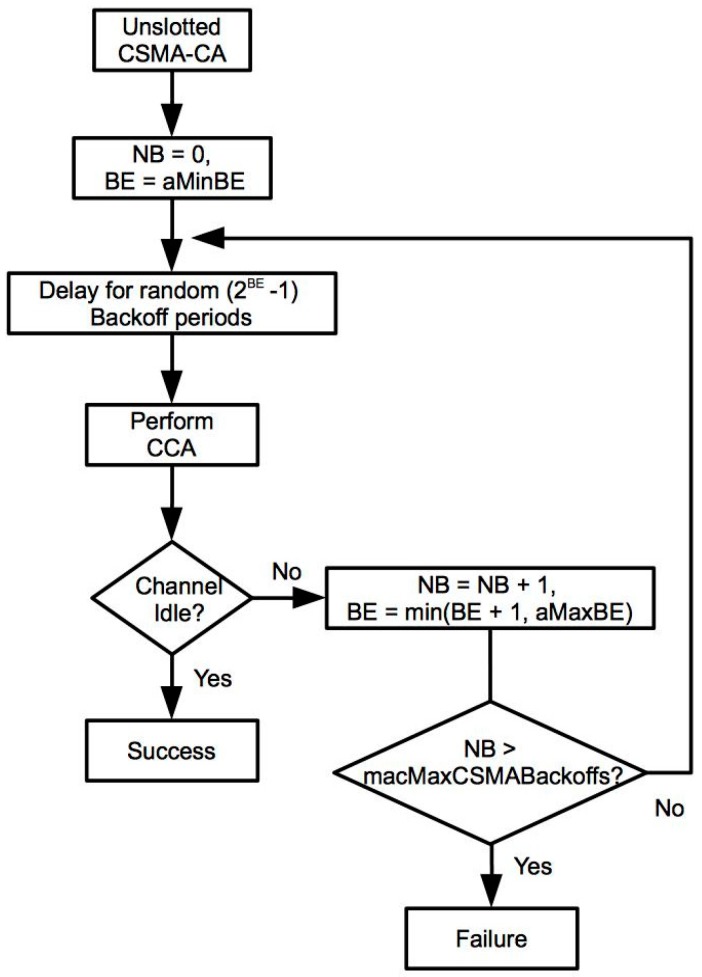
Unslotted version of the IEEE 802.15.4 CSMA-CA protocol.

Considering the unslotted version of the IEEE 802.15.4 standard, the medium access algorithm follows the flowchart shown in Figure 1.

The parameters involved in this algorithm are:

- NB: number of times a mote has tried to access the channel.
- BE: The backoff exponent represents the number of backoff periods the mote must wait before accessing the channel. The minimum (aMinBE) and maximum values (aMaxBE) in the standard are 3 and 5, respectively.
- macMaxCSMABackoffs: Maximum number of channel access tries. This value is 4 by default.

The medium access process starts determining the initial backoff period that the mote has to wait before checking the channel state. This number is a random value in the interval (0 to 2^BE^ − 1). Initially, BE is 3 and the backoff period would be in the range from 0 to 7. Next, the physical layer performs a clear channel assessment (CCA). If the channel is free, the mote can start the packet transmission, otherwise the NB and BE value are increased by one. The new BE value in this last case is calculated as BE = min (3 + 1, 5), because it cannot be greater than 5. On the other hand, NB must be always lower than macMaxCSMABackoffs. If NB exceeds this limit, the transmission is cancelled and the protocol informs the upper communications layers about this fact. 

Another important period of time that must be considered during a packet transmission is the inter frame space (IFS). IFS values are related to the packet size in the IEEE 802.15.4. So, there is a short IFS (SIFS), which is applied if the packet is short and its length is lower that aMaxSIFSFrameSize (18 bytes). In contrast, the IFS becomes long IFS (LIFS) when the packet length is higher than aMaxSIFSFrameSize. 

### 2.1. Transmission Delay

In the packet transmission delay, seven different terms can be distinguished. Each one stands for a different transmission stage during the overall packet delivery process. In this way, the total delay can be divided in the following terms:

*Delay = T_Backoff_ + T_CCA_ + T_TA_ + T_packet_ + T_TA_ + T_ACK_ + T_IFS_* (1)

where these terms stand for: (a) *T_Backoff_* is the back off period; (b) *T_CCA_* is the time to perform the CCA, which it is usually 128 μs in Telosb motes [20]; (c) *T_packet_* is the packet transmission time; (d) *T_TA_* is the turn around time that allows the device to switch from transmit mode to receive mode and vice versa, this time is normally 192 μs in Telosb motes; (e) *T_ACK_* is the time for the transmission of an ACK packet; and (f) *T_IFS_* is the final delay that can be equal to the SIFS or LISF times depending on the packet length. In the case of having a transmission that does not require an acknowledgement, *T_TA_* and *T_ACK_* disappear in (1). The most significant term in the previous list of delays is *T_Backoff_*, since it cannot be known a priori due to its random nature, and because it is usually greater than the rest of terms. The backoff period can be formulated as:

*T_Backoff_ = BO_slots_ · T_BOslots_* (2)

where the first term *BO_slots_* is the random number of backoff slots, which the mote includes before the packet transmission, and *T_BOslots_* is the duration of one slot. The number *BO_slots_* is a random value uniformly selected in the interval (0 to 2^BE^ − 1) and the initial mean value of this term is 3.5. 

In addition to all the delays involved in a packet transmission, there is another contribution coming from the operating system (OS). Thus, when a packet is sent (received), the OS must process the packet and forward (receive) the information to (from) the final application. Therefore, two additional delays appear at the beginning and the end of the delay expressed in (1). These delays are: (a) send time at the transmitter required to assemble the message and give the transmission order to the MAC layer and (b) reception time at the receiver to process the message and forward this information to the destination application.

### 2.2. Performance Simulation

Localization algorithms require the exchange of many data packets with the infrastructure beacons to get the necessary RSSI values. In this context, the assessment of the IEEE 802.15.4 networks performance is important to know the maximum bandwidth and packet exchange rate that can be achieved. In this experiment, the maximum network throughput is determined using the Cooja [21] simulator and TinyOS [22] components running on Telosb motes. The network topology considered is a star, where the receiver and all the senders are in the same broadcast domain and the packet length is fixed and equal to 23 bytes. The senders are continuously sending packets to the receiver at a constant transmission rate. Since all of the motes share the same channel and they are in the same broadcast domain, packets collisions may occur. The CSMA implementation for the CC2420 transceiver in the TinyOS distribution differs slightly from the standard version. In this case, the backoff slots are selected using a random number (*rand*) and calculating:

*BO_slots_ = rand mod (31 · CC2420_BACKOFF_PERIOD) + CC2420_MIN_BACKOFF* (3)

By default, the constants *CC2420_BACKOFF_PERIOD* and *CC2420_MIN_BACKOFF* are both equal to 10. Since *T_BOslots_* is measured with a 32 KHz timer, *T_Backoff_* is in the range from 0.32 to 10 ms. If the channel is busy, the back-off delay changes and it is computed as:

*BO_slots_ = rand mod (7 · CC2420_BACKOFF_PERIOD) + CC2420_MIN_BACKOFF* (4)

**Figure 2:**
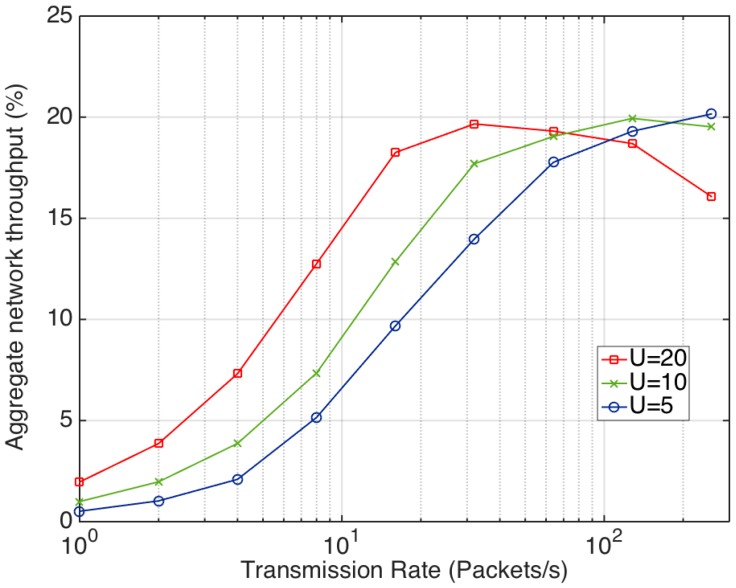
Aggregate network throughput for different transmission rates and number of nodes (*U*). The aggregate network throughput is defined as the percentage of time in which the channel is occupied with a successful packet transmission.

With values that are in the interval from 0.3125 to 2.5 ms. In these conditions, the transmission follows the unslotted CSMA algorithm depicted in Figure 1. The evaluation conducted estimates the aggregate network throughput defined as the percentage of time in which the channel is occupied with a successful packet transmission. Figure 2 shows the obtained results with three different numbers of senders and packet transmission rates. As it can be seen, initially when the number of senders increases, the network throughput also rises, especially for low transmission rates. However, the throughput decreases when the transmission rate increases too much due to packet collisions, especially when the number of senders is high. In any case, it should be noticed that none of the simulated scenarios exceeds the 25% of the network throughput. As a result, it can be concluded that the basic CSMA protocol is not suitable for applications that require high transmission rates with multiple users trying to communicate with the same receiver. 

## 3. New MAC protocol

### 3.1. Initial Assumptions 

As it was stated in the previous section, the standard CSMA protocol is not appropriate in localization systems with many users and beacons, where users have to acquire RSSI values at both sides (beacon and user mote), with multiple power levels and different channels. Consequently, the new proposed MAC protocol has been conceived to speed up this process eliminating the contention periods in the channel access. As it has been mentioned, the collection of the RSSI values between a user and a beacon comprises a sequence of packets exchanges using several frequency channels and power levels. In this context, a packets exchange is defined as the consecutive transmission of two packets in opposite directions (one from user to beacon and another from beacon to user). In this way, this sequence of packets exchanges at different channels and power levels represents a burst transmission, since it includes a continuous sequence of multiple exchanges that occupies the medium for a certain period of time. Next, the proposal of this new access protocol is carried out following the next assumptions:

- There is a channel, denoted Ch1, which is used for signaling purposes.
- Beacons announce their availability using broadcast packets.
- Users receiving these packets can reserve the beacon for subsequent transmissions using the request to send and clear to send (RTS/CTS) mechanism.
- The sequence of transmissions, including the list of channels and power levels that are used during the packet exchange with a beacon, is predefined and fixed beforehand, and all users follow the same sequence.
- The total number of packets exchanged to collect the RSSI values depends on three factors: (a) number of frequency channels tested (denoted as *K*), (b) number of different power levels considered (*L*), and (c) retransmissions for a fixed combination of a channel and a power level (*N*). Thus, the total number of packets exchanged is: 2·*N*·*L*·*K*.
- After receiving a RTS or CTS packet, other beacons and users stop using the radio during a certain time. This period of time is long enough to allow the user that has reserved the beacon to exchange all the packets at the first channel.
- A user and a beacon have a fixed period to complete the packet exchanging process at every different channel. When this period ends, they have to change from the current channel to the next.

The number of channels, power levels and retransmissions are fixed, being values configured during the system initialization. These values provide a trade-off between the localization precision and the system delay needed to collect the RSSI samples, since in general with more information a higher precision can be achieved.

### 3.2. Protocol Proposal

**Figure 3:**
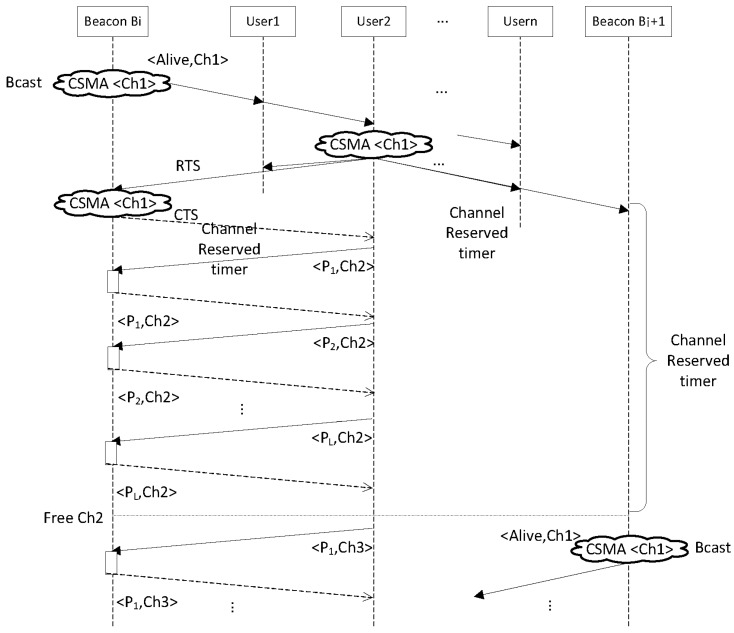
Sequence of user selection and channel access in the proposed protocol. In the figure, Ch1 identifies the frequency channel 1, Ch2 denotes channel 2 and so on. P_i_ indicates a packet transmission with this power level. RTS and CTS are the packets transmitted to establish the handshake and Alive is the broadcast packet sent by a beacon to announce its availability. When a packet transmission is marked with the CSMA term means that the mote has to implement this medium access. Channel Reserved time is the period of time in which an user and a beacon exchange all the packets at a certain frequency channel, after the expiration of this time they has to go to the next channel.

The considered scenario includes a specific number of users and infrastructure beacons that broadcast periodically packets showing their availability. The first user that reserves the beacon using a RTS/CTS packet exchange gains the channel access during a period of time that is long enough to send and receive *N* packets at every power level *L*, at the first channel of the group of *K* channels selected. This process can be seen in Figure 3, where a possible example is presented. In this case, beacon Bi announces its availability transmitting broadcast packets at channel Ch1. This is represented in Figure 3 with the symbol (CSMA <Ch1>) and the packet <Alive,Ch1>, which indicates the transmission of the packet Alive using the CSMA protocol. After the reception of this broadcast packet, User2 reserves the beacon Bi using the RTS/CTS mechanism. This process involves the transmission of two packets RTS and CTS using the CSMA protocol. All the rest of users and beacons that have received the RTS or CTS packets start a timer with an overflow time equal to the Channel Reserved time. During this period, beacons stop the transmissions of broadcast packets because the channel is occupied. 

Next, User2 starts the exchanging process of packets with the beacon Bi using channel Ch2. The sequence starts with a power level P_1_ (<P_1_,Ch2> in Figure 3) and finishes with a power level P_L_. Once beacon Bi and User2 have exchanged the required packets for all the power levels (from P_1_ to P_L_), they pass to channel Ch3 and start a similar sequence. At this instant, the Channel Reserved timers fire and, since Ch2 has been released, other beacons (for example Bi + 1 in Figure 3) can resume the announcement of their availability using Ch1.

During the packet exchange process with the beacon Bi at the different channels Ch2, Ch3, …, Chk, User2 can access the channel without having to implement the CSMA protocol, since none of the rest of users or beacons located in the same radio range can transmit during this period. Once the Users2 finishes the transmission at the first channel Ch2, and the Channel Reserved time is over, the rest of users and beacons restart their radio transceivers. If any other user finds a free beacon that is prepared to exchange packets (for example beacon Bi + 1 in Figure 3), after the RTS/CTS handshake they can start the transmission in channel Ch2, since the first user (User2) has already released it. Therefore, the protocol takes advantage of the frequency multiplexing capability of the system when several channels have to be tested, allowing that different users exchange testing packets with the corresponding beacons in parallel. But, to keep the complexity of the protocol low, the sequence in which the channels are tested is always the same. 

**Figure 4:**
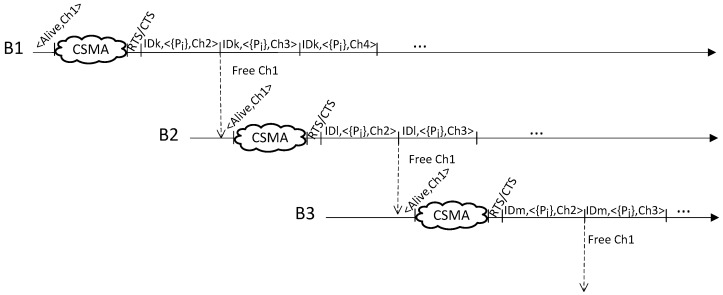
Channel access sequences (Ch1, Ch2, Ch3 and Ch4) for three users (IDk, IDl and IDm) exchanging packets with three beacons (B1, B2 and B3) in parallel. Figure shows how the packets exchanges between different pairs of users and beacons can be multiplexed in different frequency channels.

The channel access sequences for several users exchanging packets with different beacons can be seen in Figure 4. It should be noticed that a user and a beacon have a fixed period to complete the packet exchanging process at every different channel and they must change from the current channel to the next whenever this period ends. 

In Figure 4, the user IDk exchanges packets with beacon B1 using channel Ch2 for all the power levels considered in the location process, {P_j_}. Once user IDk has finished the sequence at Ch2, this channel is released and it could be used by other users. In this case, beacon B2 initiates the announcement of its availability transmitting CSMA packets using Ch1, while beacon B1continues exchanging packets with user IDk, now at channel Ch3. After the RTS/CTS handshake, IDl begins exchanging packets with beacon B2 starting at channel Ch2 and following the same sequence of channels that B1 and IDk. As it can be seen in Figure 4, our protocol allows that different pairs of users and beacons perform simultaneous transmissions of packets at different channels.

With the avoidance of the contention period during the transmission of every testing packet, the total RSSI collection delay decreases significantly. As it was stated in the previous section, for applications programmed with TinyOS the worst case of the *T_Backoff_* is 10 ms and its average value is approximately 5 ms. Reducing this delay the protocol can speed up the RSSI collection process and the location estimation. Indeed, this fact may be very important in tracking applications, where the response time of the system may impose a strict refresh time.

### 3.3. Energy Consumption Analysis 

Energy consumption is a central problem in the design of WSNs. These networks are frequently deployed outdoors, in places where accessing the mote to replace the batteries is difficult. In these scenarios, the network operation can be extended reducing the mote energy consumption, which usually leads to the implementation of some duty cycle in the radio operation. The main drawback of this solution is that normally causes an increase of the transmission delay, since both motes (emitter and receiver) have to wait and coordinate the operation periods of their wireless transceivers to perform the transmission. However, localization systems present some differences in this respect. Thus, although the underlying infrastructure is also based on WSN motes, they are mainly deployed indoors and the network motes can be easily accessed to replace their batteries. Moreover, the system beacons, which are part of the network infrastructure, are placed in fixed positions and they could be connected to the mains supply. On the other hand, since the principal objective of the proposed MAC protocol is the reduction of the RSSI collection delay, the inclusion of a radio duty cycle is not advisable. 

Consequently, the proposed MAC protocol keeps permanently the wireless transceiver in reception state. Only when a packet is transmitted, the protocol changes the transceiver state from reception to transmission. With this configuration of the radio chip, the energy consumption will be higher compared to any other MAC layer protocol that establishes a duty cycle in the radio operation, but keeping constantly the radio in the listening state significantly decreases the transmission delay. In addition, the energy consumption increase is not so critical in indoor localization systems, since mobile users can replace their motes batteries with ease and static beacons can be connected to the mains supply. 

The energy consumption of the user mote can be determined considering the periods of time in which the mote is in reception or in transmission. It is assumed that by default the mote is in reception mode, unless the mote is transmitting a packet. In this last case, the power consumption changes accordingly to the power level configured in the packet transmission. Hence, the energy consumption of the user mote for gathering all the RSSI values can be expressed as:

(5) $$E=\sum_{B}\left(\left(T_{d}-\sum_{L}T_{txL}\right)\cdot P_{rx}+\sum_{L}T_{txL}\cdot P_{txL}\right)$$

where the previous terms are: (a) *T_d_* is the total delay in the RSSI collection process, (b) *T_txL_* is the total time transmitting packets with power level *L*, (c) *P_rx_* is the mote power consumption at reception state, (d) *P_txL_* is the mote power consumption at transmission state with power level *L*, and (e) *B* is the number of beacons that are tested in the RSSI collection process.

## 4. Results

### 4.1. Simulation Results

The first evaluation considers the estimation of the total delay needed to collect all the RSSI samples. The simulated scenario, implemented in the Cooja simulator, comprises one beacon and one user that exchange packets to acquire all the RSSI values. Both elements were implemented using Telosb motes and the software was programmed with TinyOS. In the experiments, different numbers of channels and power levels in the RSSI sampling process have been considered. In contrast, the number of retransmissions remained fixed (*N* = 10). It should be noticed that the packets exchange process always entails the transmission of two packets in opposite directions, i.e., a first packet from the user mote to the beacon and a second packet from the beacon to the user mote. Thus, the total number of packets that are transmitted in both directions is:

(6) Total Number of Packets=2·N·K·L·B

where the factor 2 comes from the two packets transmitted in both directions, *N* is the number of retransmissions, *K* is the number of channels, *L* is the number of power levels and *B* is the number of beacons. For example, when *N* = 10, *L* = 5, *K* = 5 and *B* = 1, the total the number of packets is 500. In the application programmed with TinyOS the send time, the received time, the packet time and the average backoff time were experimentally measured and their values were 2.2, 1.8, 0.9 and 5.1 ms respectively. The addition of these values with the rest of terms in Equation (1) gives the total delay in the transmission and reception of a packet that is approximately 10 ms. This delay multiplied by the number of packets provides an estimation of the total delay required to collect all the RSSI samples. For the previous example, the total delay is in the order of five seconds. 

**Figure 5:**
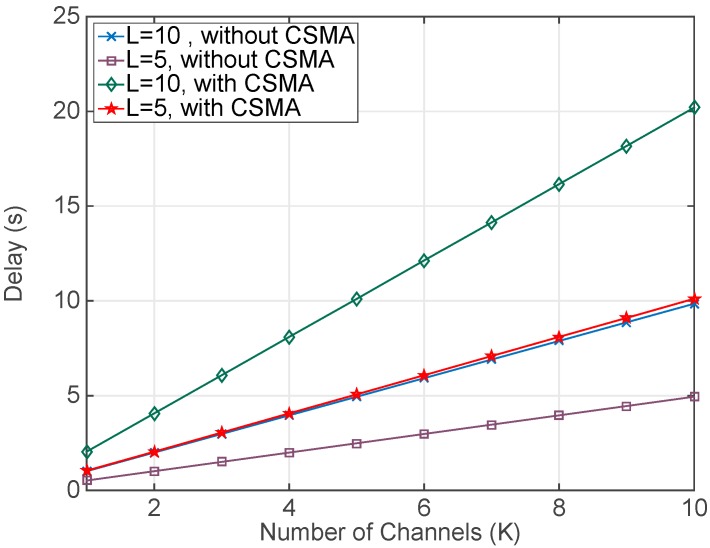
Delay to collect the RSSI values with different number of channels (*K*) and power levels (*L*). The number of retransmissions (*N*) at every combination of channel and power level is fixed and equal to 10.

Results in Figure 5 compare two cases: (a) when the motes run the proposed new MAC protocol, denoted as without CSMA, and (b) when the motes run the standard MAC protocol, described as with CSMA. As it can be seen in Figure 5, the proposed protocol without CSMA shortens significantly the total collection delay compared to the case with CSMA and divides this time by two. This is due to the TinyOS implementation of the CSMA that includes an average backoff time that is approximately equal to the sum of all the rest of delays involved in a packet transmission. Thus, results in this Figure 5 demonstrate the total delay reduction that can be achieved with the proposed MAC protocol. 

**Figure 6:**
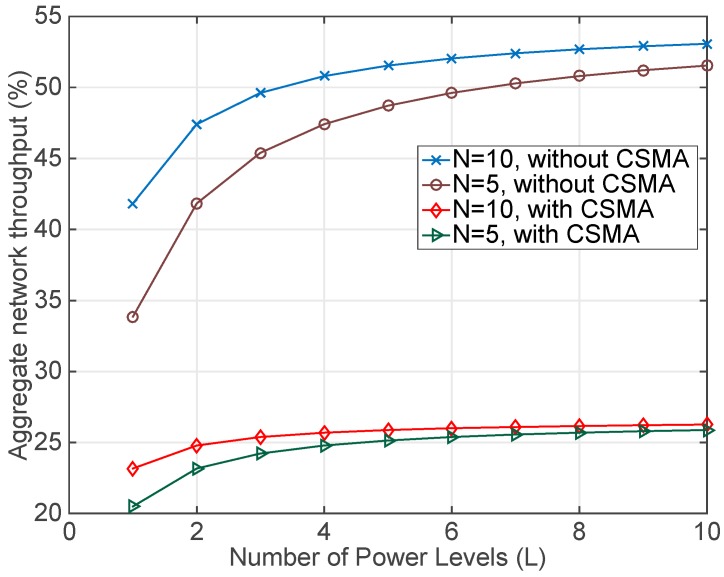
Aggregate network throughput with different power levels (*L*) and number of retransmissions (*N*) (10 or 5). The number of channels is fixed (*K* = 1) for all the combinations of *L* and *N*. The aggregate network throughput is defined as the percentage of time in which the channel is occupied with a successful packet transmission.

The second assessment conducted determines the aggregate network throughput achieved during the packet exchange process. Results are obtained using the same previous setup and simulating the collection process using only one channel (*K* = 1). Figure 6 depicts that the elimination of the CSMA increases the network throughput because less time is wasted during the channel access. Thus, comparing both cases it can be noticed that the network throughput with the new protocol duplicates the throughput obtained in the CSMA case. However, since the first stage of the channel reservation (based on the RTS/CTS handshake) still uses CSMA, the impact of this part is reflected in the overall result. Even so, the influence of this stage in the total result decreases when more packets and more power levels are added to the RSSI data gathering. 

Finally, the last assessment presented deals with the energy consumption of the proposed MAC protocol. The considered scenario is the same that in the previous cases and the energy analysis takes into account the current consumption of the CC2420 wireless transceiver [23] at each different operating state. These values are shown in Table 1. 

**Figure 7:**
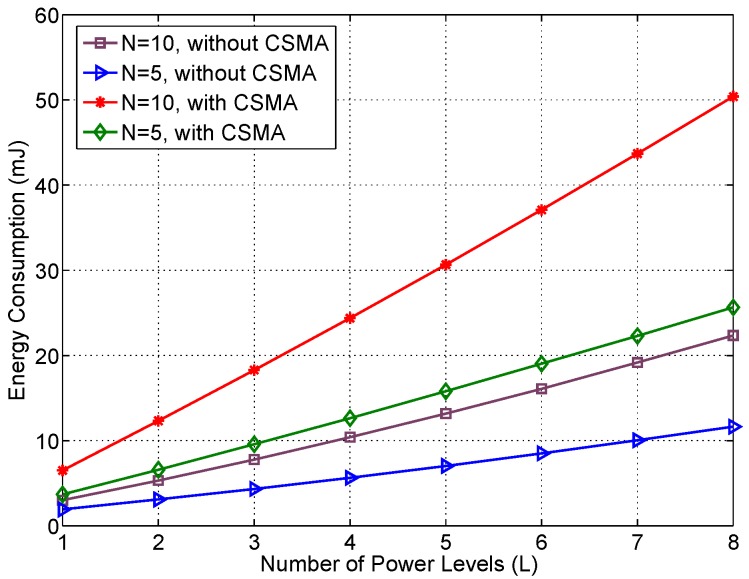
Energy consumption for different number of power levels (*L*). The number of frequency channels (*K* = 1) and beacons (*B* = 1) remained fixed. There were two numbers of retransmissions (*N* = 5) or (*N* = 10) and 8 power levels: (−25 dBm, −15 dBm, −10 dBm, −7 dBm, −5 dBm, −3 dBm, −1 dBm, 0 dBm). Power levels are added in order, i.e., the case with only one power level uses −25 dBm, the case with two power levels includes transmissions with −25 and −15 dBm and so on.

The energy consumption analysis focuses on the wireless transceiver because it is by far the component with the highest consumption in a Telosb mote. Assuming that the wireless transceiver has a power supply of 1.8V and applying Equation (5), the total energy consumption for transmitting and receiving all the RSSI values can be calculated. Figure 7 presents the obtained results, as it can be seen the new MAC protocol without CSMA outperforms the CSMA case, due to the reduction of the listening intervals caused by the elimination of the contention periods. It is also significant the increase of the energy consumption with the number of retransmissions because the total number of packets and the total delay in the RSSI collection process rise.

### 4.2. Experimental Results

**Figure 8:**
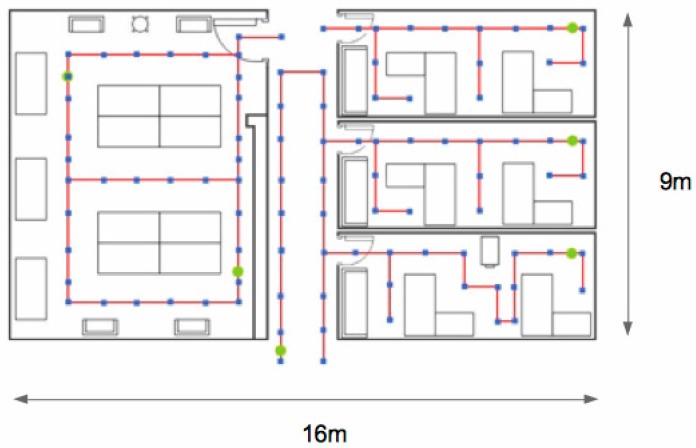
Covered area in the experimental setup deployed in our facilities. The scenario covers three offices, a laboratory and part of the main corridor.

The proposed protocol has been evaluated experimentally in our facilities. The experimental setup includes an area consisting of three offices, a large laboratory and part of the main corridor. The considered scenario covers a rectangular box-shaped area of dimensions 9 m × 16 m, which is divided by walls and contains furniture, as it is shown in Figure 8. 

**Figure 9:**
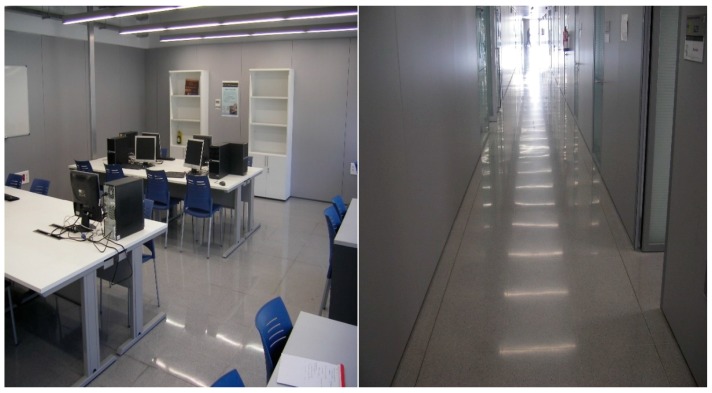
Pictures showing the laboratory and the main corridor included in the testing area.

The setup also comprises 6 beacons that were deployed at different offices, in the laboratory and the corridor. The specific location of every beacon is represented by a green spot in Figure 8, besides the red line shows the path followed by a robot that was used to collect the RSSI values at the blue spots. Figure 9 includes two pictures showing the laboratory and the main corridor.

The beacons used in the setup were constituted using Telosb motes. The robot was connected to an additional Telosb mote placed on a structure assembled on the robot that was a meter high to avoid the ground effect in the signal transmission. The collection process has involved the sampling of RSSI values at every testing point (blue points in Figure 8) exchanging packets with 6 beacons at both sides (two packets were sent in opposite directions to collect the RSSI values at the beacon and at the robot). In addition, different frequency channels and power levels were used during the gathering of the RSSI values at every testing point.

Once the database was completed, a second round started to collect new RSSI values for localizing one user in the setup area. During this step a new sequence of RSSI values were taken at the testing points. With these values, the position of the user was estimated using the localization algorithm presented in [24]. The main feature of this algorithm is its capacity to take advantage of all the information that can be drawn when multiple channels and power levels are used during the RSSI sampling process. This additional information can improve the localization precision at the expense of increasing the effort needed to collect the RSSI values. Additionally, the localization time is also increased because the user has to transmit and receive more packets to gather all the RSSI values at different channels and power levels. In this context, the new proposed MAC protocol can effectively reduce the localization time eliminating the contention periods when a large amount of RSSI values taken at different channels and power levels have to be collected.

**Figure 10:**
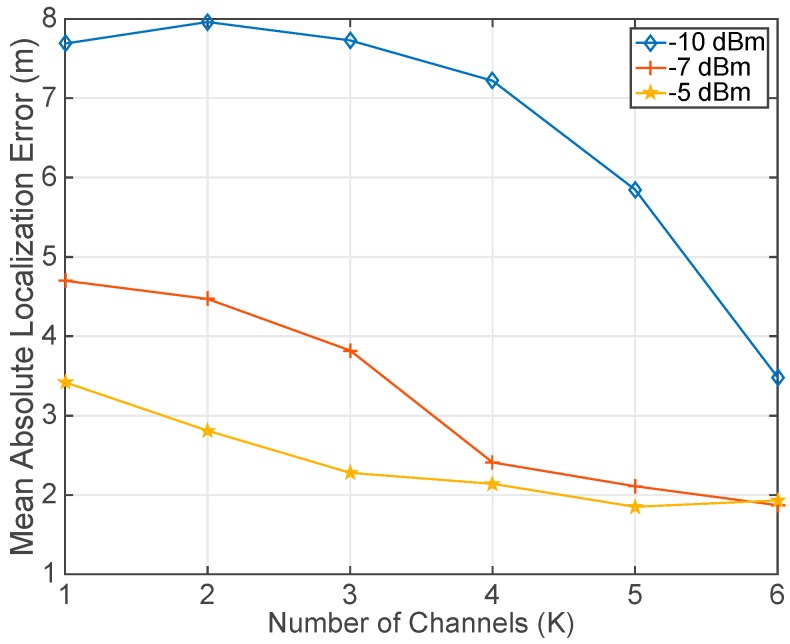
Mean Absolute Localization Error for different numbers of frequency channels (*K*). The number of retransmissions (*N* = 5), power levels (*K* = 1) and beacons (*B* = 6) remained fixed for each different line in the graph. Three lines are presented according to three different power levels: (−10 dBm, −7 dBm, −5 dBm). Channels used are: [11 (2405 MHz), 13 (2415 MHz), 16 (2430 MHz), 19 (2445 MHz), 22 (2460 MHz), 26 (2480 MHz)]. Channels are added in order, i.e., the case with only one channel uses channel 11, the case with two channels includes channels 11 and 13, and so on. Localization was carried out after transmitting and receiving all the packets exchanged between one user and six beacons.

The experimental evaluation began considering the impact that the number of channels (*K*) has on the localization precision. To this end, the mean absolute localization error was calculated placing the user at every testing point. The user gathered the RSSI values exchanging packets with the 6 beacons (*B* = 6) and repeating the transmission 5 times (*N* = 5). The position estimation is based on the algorithm presented in [8,24]. In this experiment different power levels (*L*) and numbers of channels (*K*) were considered to study their effect in the localization precision. Figure 10 presents the error obtained testing only one power level and different numbers of frequency channels. As it can be noticed in Figure 10, the increase of the number of channels improves the location precision regardless of the power level. An additional conclusion is that in our scenario due to the diversity of obstacles that prevent having direct line of sight between the user and the beacons, higher power levels provide better location accuracy because with these levels larger radio ranges can be covered.

**Figure 11:**
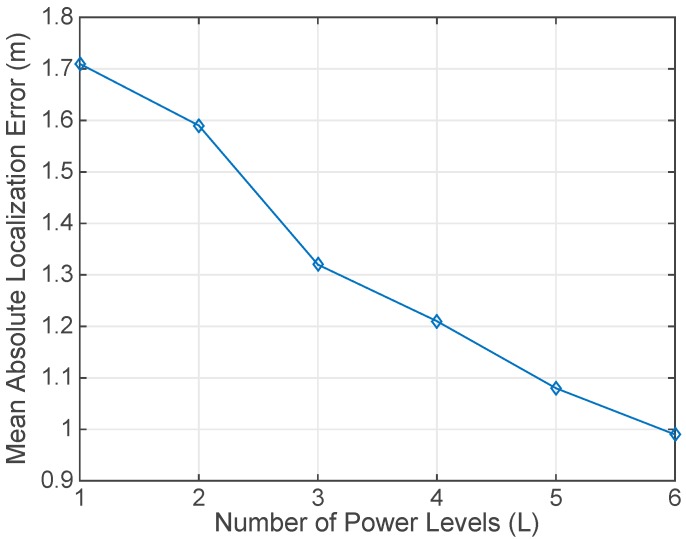
Mean Absolute Localization Error for different number of power levels (*L*). The number of retransmissions (*N* = 5), frequency channels (*K* = 6) and beacons (*B* = 6) remained fixed. Channels were: [11 (2405 MHz), 13 (2415 MHz), 16 (2430 MHz), 19 (2445 MHz), 22 (2460 MHz), 26 (2480 MHz)]. There were 6 power levels considered: (0 dBm, −1 dBm, −3 dBm, −5 dBm, −7 dBm, −10 dBm). Power levels are added in order, i.e., the case with only one power level uses 0dBm level, the case with two power levels includes transmissions with 0 and −1 dBm, and so on. Localization was carried out after transmitting and receiving all the packets exchanged between one user and 6 beacons.

The second experiment was focused on the effect of adding more power levels (*L*). In this case, at every testing point the user exchanged packets using 6 frequency channels (*K* = 6) and a sequence of different power levels (*L* = [1 to 6]) with 5 retransmissions (*N* = 5). Results presented in Figure 11 show that the addition of more RSSI values collected at different power levels improves the localization precision. 

However, the improvement in the location accuracy is achieved at the expense of the increase in the number of packets exchanged, which causes longer delays in the location time. It should be noticed that results in Figure 10 and Figure 11 are obtained regardless of the MAC protocol implemented in the motes. This is because the MAC protocol can change the total collection delay, which is not represented in these graphs, but the acquired RSSI values are the same independently of the protocol. As a result, the localization algorithm will estimate the same position and both protocols will provide the same localization error. These two experiments have been included to highlight the benefits of including more channels and power levels in the localization accuracy. However, the addition of more channels and power levels increases the collection delay. In this sense, the application of the proposed MAC protocol diminishes this effect and allows a higher localization precision when a maximum delay in the RSSI collection process is established. 

**Figure 12:**
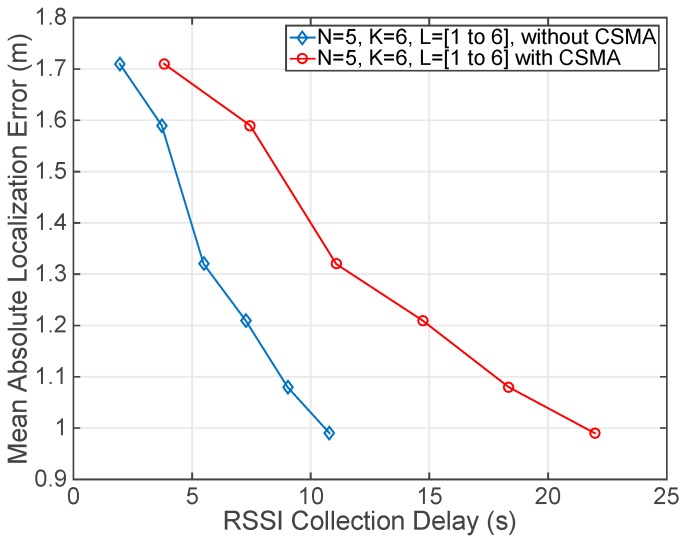
Mean Absolute Localization Error against RSSI collection delay. The number of retransmissions (*N* = 5), frequency channels (*K* = 6) and beacons (*B* = 6) remained fixed. Channels were: [11 (2405 MHz), 13 (2415 MHz), 16 (2430 MHz), 19 (2445 MHz), 22 (2460 MHz), 26 (2480 MHz)]. There were 6 power levels considered: (0 dBm, −1 dBm, −3 dBm, −5 dBm, −7 dBm, −10 dBm). Power levels were added in order. Delays were measured after transmitting and receiving all the involved packets with only one user and six beacons.

The last experiment relates the location precision to the RSSI collection delay. The setup is the same of the previous case and was conducted in our real deployment. In this experiment, the number of frequency channels (*K* = 6), retransmissions (*N* = 5) and number of beacons (*B* = 6) remained fixed. In contrast, the number of power levels varied from 1 to 6 (*L* = [1 to 6]). As it can be seen in Figure 12, the obtained results establish a trade-off between the delay in the RSSI collection process to perform the localization of the user, at one specific point in the considered scenario, and the location precision that can be achieved. In this way, the new proposed MAC protocol allows the reduction of the RSSI collection delay eliminating the contention periods and enabling the concurrent transmission of several users at different frequency channels. As it is proved in Figure 12, the new MAC protocol outperforms the MAC protocol proposed in the IEEE 802.15.4 standard and divides by two the time needed to collect all the RSSI values for a specific combination of *K*, *N*, *B* and *L*, which establishes the localization precision. Thus, it can be concluded that the proposed algorithm reduces by two the localization delay for a certain localization precision.

## 5. Conclusions

In this article, a MAC protocol adapted specifically to the RSSI collection process for indoor location systems based on IEEE 802.15.4 networks, with multiple power levels and frequency channels, is presented. Its main advantage over other state-of-the-art approaches is that it makes use of different frequency channels to avoid the medium access contention periods, without having to use complex scheduling algorithms or tight clock synchronization protocols. The proposed protocol establishes an initial RTS/CTS mechanism to reserve a beacon and the access to the medium. Once a user gains the medium access, it has a channel-reserved period to exchange all the required packets at the first channel without any interference from the rest of users or beacons. This fact allows the elimination of contention periods during the subsequent packet exchange process. Other users can overlap their packet exchange processes with different beacons using channels that have been tested and released by previous users. The conducted experiments have included both simulated results and the localization of one user in a real deployment. Simulated results show the benefits of eliminating the contention periods in terms of the total RSSI collection delay and network throughput. In addition, the new protocol proposed is rather simple and it can be easily implemented in WSNs motes with limited resources. Experimental results obtained in a real deployment with Telosb motes have proved the suitability of the new MAC protocol for reducing the RSSI collection delay. In addition, the real deployment results provide a trade-off between the location precision and the RSSI collection delay when multiple channels and power levels are added.

## Figures and Tables

**Table 1:** Current consumption of the CC2420 wireless transceiver at reception and transmission modes with different power levels. Values are taken from the CC2420 datasheet [23].

Mode	Current Consumption
Reception	18.8 mA
Transmission (*L* = −25 dBm)	8.5 mA
Transmission (*L* = −15 dBm)	9.9 mA
Transmission (*L* = −10 dBm)	11.2 mA
Transmission (*L* = −7 dBm)	12.5 mA
Transmission (*L* = −5 dBm)	13.9 mA
Transmission (*L* = −3 dBm)	15.2 mA
Transmission (*L* = −1 dBm)	16.5 mA
Transmission (*L* = 0 dBm)	17.4 mA
